# Lumbar microdiscectomy for sciatica in adolescents: a multicentre observational registry-based study

**DOI:** 10.1007/s00701-017-3077-4

**Published:** 2017-01-16

**Authors:** Sasha Gulati, Mattis A. Madsbu, Tore K. Solberg, Andreas Sørlie, Charalampis Giannadakis, Marius K. Skram, Øystein P. Nygaard, Asgeir S. Jakola

**Affiliations:** 1 Department of Neurosurgery, St. Olavs University Hospital, Trondheim, Norway; 2 Department of Neuroscience, Norwegian University of Science and Technology (NTNU), Trondheim, Norway; 3 National Advisory Unit on Spinal Surgery, St. Olavs University Hospital, Trondheim, Norway; 4 Department of Neurosurgery, University Hospital of Northern Norway (UNN), Tromsø, Norway; 5 The Norwegian National Registry for Spine Surgery, University Hospital of Northern Norway (UNN), Tromsø, Norway; 6 Department of Medicine, Ålesund Hospital, Ålesund, Norway; 7 Department of Paediatrics, Rikshospitalet, Oslo University Hospital, Oslo, Norway; 8 Department of Neurosurgery, Sahlgrenska University Hospital, Blå Stråket 5, vån 3, 413 45 Göteborg, Sweden; 9 Institute of Neuroscience and Physiology, Sahlgrenska Academy, Gothenburg, Sweden

**Keywords:** Adolescent, Disc herniation, Microdiscectomy, Spine

## Abstract

**Background:**

Lumbar disc herniation (LDH) is rare in the adolescent population. Factors predisposing to LDH in adolescents differ from adults with more cases being related to trauma or structural malformations. Further, there are limited data on patient-reported outcomes after lumbar microdiscectomy in adolescents. Our aim was to compare clinical outcomes at 1 year following single-level lumbar microdiscectomy in adolescents (13–19 years old) compared to younger adults (20–50 years old) with LDH.

**Methods:**

Data were collected through the Norwegian Registry for Spine Surgery. Patients were eligible if they had radiculopathy due to LDH, underwent single-level lumbar microdiscectomy between January 2007 and May 2014, and were between 13 and 50 years old at time of surgery. The primary endpoint was change in Oswestry Disability Index (ODI) 1 year after surgery. Secondary endpoints were generic quality of life (EuroQol five dimensions [EQ-5D]), back pain numerical rating scale (NRS), leg pain NRS and complications.

**Results:**

A total of 3,245 patients were included (97 patients 13–19 years old and 3,148 patients 20–50 years old). A significant improvement in ODI was observed for the whole population, but there was no difference between groups (0.6; 95% CI, −4.5 to 5.8; *p* = 0.811). There were no differences between groups concerning EQ-5D (−0.04; 95% CI, −0.15 to 0.07; *p* = 0.442), back pain NRS (−0.4; 95% CI, −1.2 to 0.4; *p* = 0.279), leg pain NRS (−0.4; 95% CI, −1.2 to 0.5; *p* = 0.374) or perioperative complications (1.0% for adolescents, 5.1% for adults, *p* = 0.072).

**Conclusions:**

The effectiveness and safety of single-level microdiscectomy are similar in adolescents and the adult population at 1-year follow-up.

## Introduction

The lumbosacral radicular syndrome, also known as sciatica, is commonly caused by a herniated disc [11]. In the majority of patients the natural course of sciatica is favourable [21]. The international consensus is that surgical treatment is offered if the radiating leg pain persists despite a period of conservative management [1]. In the adolescent population lumbar disc herniation (LDH) is rare, but when present it generally causes symptoms similar to those in the adult population [6]. In adults the outcome after surgical treatment of lumbar disc herniation with lumbar microdiscectomy is well established and favourable [15, 16, 20], but treatment and outcome are less well defined in adolescents [6].

There seems to be different predisposing factors in the adolescent populations with LDH compared to the adult population. Dang et al. [5] reported recently that spinal malformations were more common in the adolescents, but outcome was not improved by fusion. Another factor causing LDH seen more often in adolescents is trauma [6] and, since adolescents have less widespread degeneration, the outcome may be better than in adults, as recently reported [12]. In adolescents the growing spine may also have an impact on outcome, and adolescents may have different demands and expectations with respect to outcome compared to their peers and compared to the adult population. Consequently, outcome studies with implementation of patient reported data in adolescent LDH patients are needed.

There are limited data on patient-reported outcomes after lumbar microdiscectomy in adolescents, and the literature consist largely of small, retrospective series [6]. To achieve adequate patient numbers to study rare entities and subgroups, such as adolescents, LDH spine registries are invaluable.

In fact, a recent study from the Swedish Spine Registry (SweSpine) demonstrated that adolescents were more satisfied and had fewer spine-related symptoms following surgery than adult patients [12]. The SweSpine study included patients that were operated on with both open discectomies and microdiscectomies [12, 19]. As there is only one prospective study on surgical management of LDH in the adolescent population, there is a need to validate the results in another population to establish the effectiveness of treatment with high external validity [12]. As microdiscectomy is more common than open discectomies in the adolescent population, a more focused study is necessary to evaluate effectiveness of this particular procedure.

Due to the differences in aetiology and the sparse patient reported outcome data in the adolescent LDH population, further data are needed to assess treatment effectiveness in terms of patient reported outcomes and safety. We hypothesised that outcome in adolescents would be better compared to adults due to the more focal disease; thus, surgery, being a focal treatment, would be more targeted.

The primary aim of this registry-based study was to compare functional results at 1 year after single-level lumbar microdiscectomy in adolescents (13–19 years old) and younger adults (20–50 years old) with LDH using data from the Norwegian Registry for Spine Surgery (NORspine).

## Patients and methods

### Study population

Data for this observational study were collected through NORspine, a comprehensive registry for quality control and research. In total, 36 of 40 centres performing lumbar spine surgery in Norway report to NORspine. NORspine is linked to the National Registry and Statistics Norway, which contain information concerning everyone who either is or has been a resident in Norway. According to the Norwegian Directorate of Health, approximately 65% of all patients who undergo lumbar spine surgery in Norway are included in NORspine [13]. Participation in the registration by providers or patients was not mandatory, nor was participation required as a necessary condition for a patient to gain access to healthcare or for a provider to be eligible for payment. Follow-up time from the date of the operation was 1 year. Follow-up time from the date of the operation (baseline) was 1 year.

### Inclusion criteria

1. Diagnosis of sciatica due to LDH
2. Scheduled operation (i.e. non-emergency surgery) with single-level lumbar microdiscectomy between January 2007 and May 2014
3. Included in the NORspine registry
4. Age at time of surgery between 13 and 50 years old

### Exclusion criteria

1. History of lumbar spine surgery
2. Extraforaminal LDH
3. Spondylolisthesis and/or scoliosis
4. Fusion surgery

### Ethical approval

The study was approved by the regional committee for medical research in Central-Norway (2016/840) and all participants provided written informed consent. The Norwegian Data Protection Authority approved the registry protocol.

### Primary outcome measure

We used version 2.0 of the Oswestry Disability Index (ODI) [8] as the primary endpoint. ODI is a widely accepted outcome measure in surgery for degenerative lumbar spine disorders, including surgery for LDH [2, 22]. This version is translated into Norwegian and has been validated for psychometric properties [9, 17]. The ODI questionnaire is used to quantify disability for degenerative conditions of the lumbar spine and covers intensity of pain, ability to lift, ability to care for oneself, ability to walk, ability to sit, sexual function, ability to stand, social life, sleep quality and ability to travel. For each topic there are six statements describing potential scenarios, and patients select the one that most closely resembles their situation. The index is scored from 0 to 100. Zero means no disability and 100 reflects maximum disability. The minimal important change (MIC) in ODI score is considered to be approximately 10 points [14].

### Secondary outcome measure

Changes in generic health-related quality of life were measured with the generic EuroQol five dimensions (EQ-5D) instrument between baseline and 1-year follow-up. Intensity of pain was graded in two separate 0–10 numerical rating scales (NRSs) for back pain and leg pain, where 0 equals no pain and 10 represents the worst imaginable or ever experienced pain by the patient [10]. The NRS pain scales and ODI have shown good validity and are frequently used in research on back pain [9]. Complications were registered as described in the paragraph below. We also compared duration of procedures, length of hospital stays, and repeated surgery at the index level within 3 months of surgery between groups.

### Data collection and registration by the NORspine registry protocol

On admission for surgery, the patients completed the baseline questionnaire, which included questions about demographics and lifestyle issues in addition to the primary and secondary outcome measure. Information about marital status, educational level, body-mass index (BMI) and tobacco smoking was available in the NORspine registry. During the hospital stay, using a standard registration form, the surgeon recorded data concerning diagnosis, previous lumbar spine surgery, comorbidity, American Society of Anesthesiologists (ASA) grade, treatment and image findings. The surgeons provided the following complications and adverse events to the NORspine registry: intraoperative haemorrhage requiring blood replacement, postoperative haematoma requiring repeated surgery, unintentional durotomy, nerve injury, cardiovascular complications, respiratory complications, anaphylactic reactions and wrong level surgery. Patients reported the following complications if they occurred within 3 months of surgery: wound infection, urinary tract infection, pneumonia, pulmonary embolism and deep vein thrombosis. A questionnaire was distributed to all patients at 3 months and 1 year after surgery. The patients who did not respond received one reminder with a new copy of the questionnaire. The patients completed preoperative questionnaire data and postal follow-up questionnaires without any assistance from the treating surgeon.

### Surgical procedures

All patients underwent single-level lumbar microdiscectomy. Since this is a multicentre observational study, small variations in the surgical management may occur and the surgical procedures can only be described in general terms and in accordance with the data collected in the NORspine registry. The microsurgical discectomy involves preoperative fluoroscopy for detection of the target level, paramedian or median incision of about 3–6 cm, straight or curved opening of the paravertebral muscular fascia, subperiosteal release of the paravertebral musculature from the spinous process and basal lamina above and below the target disc-level. Self-retaining retractors (typically Caspar retractors) and a microscope or loupes are introduced. Often a flavectomy and arcotomy of the lamina above the disc-level are done. This is followed by careful mobilisation of the dural sac and the nerve-root medially, before evacuating the herniated disc. This might involve entering the disc space, or just removing a free sequestrated disc fragment (sequestrectomy).

### Statistical analysis

Statistical analyses were performed with SPSS version 21 (IBM Corporation, Chicago, IL, USA). Statistical significance level was defined as *p* ≤ 0.05 on the basis of a two-sided hypothesis test with no adjustments made for multiple comparisons. Central tendencies are presented as means when normally distributed and as medians when skewed. We used the chi-squared test for categorical variables. Baseline and 1-year scores were compared with paired-samples *t*-test. Mean change scores between the groups were analysed with independent-samples *t*-test. A multiple linear regression model was applied to assess the relationship between the difference in ODI score at 12-months (dependent variable) and age group (adolescence versus young adults), controlling for potential confounders. The multiple linear regression analysis included BMI (linear), adolescence (yes/no), sex, smoking (yes/no), and preoperative ODI (linear).

### Missing data

For missing data we chose to exclude cases pairwise in the complete case analyses. This method excluded patients only if they were missing the data required for the specific analysis. They were still included in any of the analyses for which they had the necessary information. This strategy was based on a study on an equivalent patient population from NORspine that showed no difference in outcomes between responders and non-responders [18]. To minimise the number of missing data points, additional “last observation carried forward” analyses were also performed. In patients where the ODI score at 1 year after surgery was missing, we used the value registered at 3 months assuming little difference between these two time points.

## Results

### Study population

**Fig. 1:**
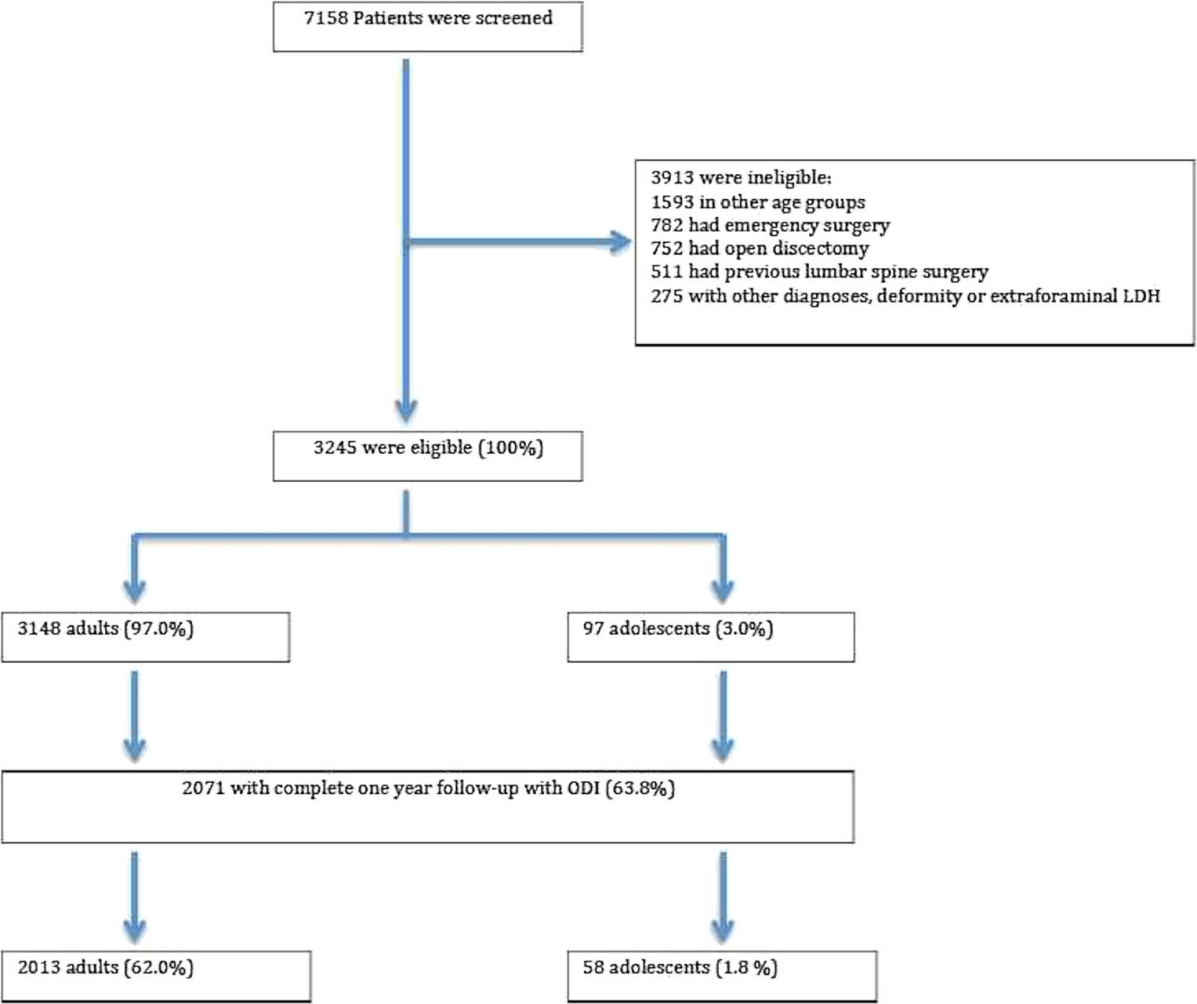
Study enrollment and follow-up

A total of 3,245 patients were enrolled out of 7,158 screened patients (Fig. 1). Among the 7,158 patients screened for inclusion, 752 underwent open discectomy and were excluded from the study. There were 97 adolescents and 3,148 adults. The mean age at baseline was 37.0 (±8.3) years and 40.7% were females. Baseline characteristics are presented in Table 1.

**Table 1:** Demographic characteristics, coexisting illnesses and measures of health status for both groups (*n* = 3,245)

Variable	Adolescents (age <20), *n* = 97	Adults (age 20–50), *n* = 3,148	*p* value
Mean age, years ± SD	17.5 ± 1.6	37.6 ± 7.7	<0.001
Female sex, no. (%)	48 (49.5)	1271 (40.4)	0.072
BMI ± SD^a^	24.4 ± 4.1	26.5 ± 4.4	<0.001
Daily tobacco smoking, no. (%)	13 (13.4)	955 (30.7)	<0.001
ASA grade >2^b^	0 (0)	47 (1.5)	0.224
Preoperative ODI ± SD^c^	33.9 ± 13.0	41.3 ± 16.6	<0.001
Preoperative EQ-5D ± SD	0.38 ± 0.34	0.34 ± 0.34	0.265
Preoperative leg pain (NRS) ± SD	6.3 ± 2.2	6.5 ± 2.2	0.525
Preoperative back pain (NRS) ± SD	5.6 ± 2.5	5.8 ± 2.3	0.333
Preoperative paresis, no. (%)	2 (2.1)	342 (10.9)	0.006
Duration of leg pain >1 year, no. (%)	28 (29.5)	720 (24.0)	0.221

^a^The body­mass index (*BMI*) is the weight in kilograms divided by the square of the height in metres

^b^The American Society of Anesthesiologists (*ASA*) grade ranges from I to V; grade V is the worst, indicating life-threating condition

^c^Oswestry Disability Index (*ODI*) ranges from 0 to 100; lower scores indicating less severe symptoms

### Oswestry Disability Index

Complete 1-year follow-up for ODI was achieved in 63.8% of patients (*n* = 2,071) with no differences between adolescents and adult patients (59.8% vs 63.9%, *p* = 0.402). For the whole study population there was an improvement of 27.2 points in ODI at 1 year (95% CI, 26.3–28.0; *p* < 0.001). In a complete case analysis (*n* = 2,071) there was no difference in mean ODI change between age cohorts at 1-year follow-up (mean difference, 0.6; 95% CI, −4.5 to 5.8; *p* = 0.811). Among the 2071 patients with complete 1-year follow-up, 82.7% (*n* = 1,711) achieved a MIC predefined as an improvement of ≥10 points in ODI score from baseline. At 1 year, 86.2% of adolescents had achieved a MIC, compared to 82.6% of adult patients (*p* = 0.474). Changes in ODI score are presented in Table 2.

**Table 2:** Primary and secondary patient reported outcomes at 1 year

Complete case analysis								
	Adolescents (*n* = 59)			Adults (*n* = 2,013)			Difference in mean change between groups (95% CI)	*p* value
	Baseline	1 year	Mean change	Baseline	1 year	Mean change		
ODI	35.0	8.5	26.5	41.3	14.1	27.2	0.6 (−4.5, 5.8)	0.811
EQ-5D	0.36	0.83	0.48	0.34	0.78	0.43	−0.04 (−0.15, 0.07)	0.442
Back pain NRS	5.7	2.2	3.5	5.7	2.7	3.1	−0.4 (−1.2, 0.4)	0.279
Leg pain NRS	6.3	1.4	4.9	6.5	2.0	4.5	−0.4 (−1.2, 0.5)	0.374
Last value carried forward analysis								
	Adolescents (*n* = 77)			Adults (*n* = 2,470)			Difference in mean change between groups (95% CI)	*P*-value
	Baseline	1 year	Mean change	Baseline	1 year	Mean change		
ODI	33.7	8.3	25.4	41.2	14.6	26.6	1.2 (−3.3, 5.8)	0.593
EQ-5D	0.38	0.85	0.47	0.34	0.77	0.43	−0.04 (−0.13, 0.05)	0.386
Back pain NRS	5.5	2.0	3.5	5.8	2.7	3.1	−0.4 (−1.1, 0.3)	0.261
Leg pain NRS	6.4	1.3	5.0	6.5	2.0	4.5	−0.5 (−1.2, 0.2)	0.173

### Secondary outcomes

Changes in EQ-5D, back pain NRS and leg pain NRS after 1-year follow-up for both age groups are presented in Table 2. No differences between the two age groups were found for any of the secondary patient-reported outcomes. Details regarding surgical treatment, duration of procedure, hospitalisation period and complications are presented in Table 3. There were no differences between groups in duration of surgery. Adolescents had slightly longer hospital stays (mean difference, 0.4 days; *p* < 0.042). Further, there were no differences in the rate of repeated surgery for any cause within 3 months between adolescents and adults (0% vs 1.2%, *p* = 0.270). The proportion of patients experiencing one or more complications within 3 months of surgery (both surgeon and patient-reported) was 5.0% (*n* = 160). There were no differences between groups in perioperative complications (i.e. during hospital admission) or complications occurring within 3 months of surgery.

**Table 3:** Surgical treatments, complications and events

Variable	Adolescents (*n* = 97)	Adults, 20–50 years (*n* = 3,148)	*p* value
Level operated, no. (%)			
- L2/L3	1 (1.0)	18 (0.6%)	0.559
- L3/L4	2 (2.1)	83 (2.6)	0.727
- L4/L5	55 (56.7)	1,235 (39.2)	0.001
- L5/S1	39 (40.2)	1,812 (57.6)	0.001
Operation time (minutes)	56.6	54.7	0.488
Days in hospital, no.	1.9	1.5	0.042
Any complication, no. (%)	1 (1.0)	159 (5.1)	0.072
Perioperative complications, no. (%)	0 (0.0)	63 (2.0)	0.159
- Dural tear or spinal fluid leak	0 (0)	35 (1.1)	0.296
- Nerve injury	0 (0)	6 (0.2)	0.667
- Blood replacement or postoperative haematoma	0 (0)	11 (0.3)	0.560
- Cardiovascular complications	0 (0)	1 (0.0)	0.861
- Respiratory complications	0 (0)	1 (0.0)	0.861
- Anaphylactic reaction	0 (0)	4 (0.1)	0.725
- Wrong level surgery	0 (0)	4 (0.1)	0.725
Complications within 3 months, no. (%)	1 (1.5)	100 (4.9)	0.214
- Wound infection	0 (0)	57 (2.8)	0.173
- Urinary tract infection	0 (0)	20 (1.0)	0.424
- Pneumonia	0 (0)	7 (0.3)	0.637
- Pulmonary embolism	0 (0)	0 (0)	–
- Deep vein thrombosis	0 (0)	0 (0)	–
- Micturition problems	1 (1.5)	24 (1.2)	0.787

### Multiple regression analysis

A multiple regression analysis was performed with difference in ODI score at 1 year as the dependent variable (Table 4). Smoking (*p* < 0.001), ASA grade >2 (*p* = 0.003), female sex (*p* = 0.010), BMI (*p* = 0.011) and preoperative ODI (*p* < 0.001) were associated with statistically significant ODI change at 1 year, whereas no association was found for adolescence (*p* = 0.103).

**Table 4:** Multiple regression analysis with change in ODI at 1 year as the dependent variable

Variable	Parameter estimate	95% confidence interval	*p* value
Adolescent	3.4	−4.5, 4.6	0.103
Oswestry score, pre-surgery	0.9	0.8, 0.9	<0.001
Smoking	−5.7	−7.2, −4.3	<0.001
ASA grade >2	−8.7	−14.5, −2.9	0.003
Female sex	−1.8	−3.1, −0.4	0.010
BMI	−0.2	−0.4, −0.05	0.011

A negative score means a worsening of ODI score 1 year after surgery

## Discussion

This multicentre observational study from NORspine shows similar effectiveness and safety of single-level lumbar microdiscectomy in adolescents and adults at 1 year. A MIC was achieved in 86% of adolescents following microdiscectomy for LDH, similar to the adult population.

Clinical outcomes after surgical treatment for LDH in the adolescent population are mostly limited to retrospective case series [6]. Our findings support the retrospective findings that this is an effective procedure, although as expected in a prospective study using patient reported outcomes, the success rates are somewhat lower than in the retrospective case series with surgeon reported outcomes [6]. In a recent observational study from SweSpine, Lagerbäck et al. [12] found that adolescents were more satisfied and had fewer spine related symptoms following surgery than adult patients. The primary outcome variable in the Swedish study was a crude self-rating of satisfaction of surgical outcomes. Our study shows that the improvements in ODI and EQ-5D at 1 year were similar in adolescents compared to the adult group. The study by Lagerbäck et al. was larger with 151 adolescent patients, but did not provide details about the surgical procedure. In a related study from SweSpine in the same time period there were 49% open discectomies [19]. However, we think that there are no reasons why spine surgeons should choose open procedures in adolescent patients if microdiscectomy offers similar improvement. Our series with 97 adolescent patients undergoing microdiscectomy is the largest prospective study to date evaluating results after minimally invasive spine surgery in this age group. Based on these two registry-based studies, it is not possible to directly compare open discectomy with microdiscectomy; however, it is interesting that the 86% satisfaction rate reported by Lagerbäck et al. compares well to the 86% of our patients achieving the MIC for ODI.

Our study seems to be consistent with previous studies showing that adolescents have less severe symptoms at baseline and that adolescents are less likely to present with paresis [6, 12]. The same trend was observed in the recent SweSpine study; however, in their study EQ-5D scores were higher (i.e. experiencing less problems) among adolescents. This is not unexpected since NRS captures pain intensity only, while the ODI and EQ-5D are multi-dimensional and focus on functional status. A clinical relevant age effect per se in EQ-5D is not expected, but spine-related or other co-morbidity may influence results [3]. Moreover, adolescents are expected to have less spinal degenerative changes at presentation.

Finally, based on the literature, it seems that surgical treatment of LDH in the adolescent population is a safe procedure with low operative complications, although open discectomies seem to be the dominating procedure [4, 12]. Our study demonstrates safety also after lumbar microdiscectomy with a complication rate of only 1%. The low complication rate in our study might be related to the young age of included patients and exclusion of individuals who had undergone previous lumbar spine surgery.

### Study strengths and limitations

The results in the present study were strengthened by the use of specific inclusion and exclusion criteria, prospective data collection and the large sample size. One of the main advantages of using data from spine registries such as NORspine is the use of widely accepted and validated outcome measures such as ODI, EQ-5D, back pain NRS and leg pain NRS. The preoperative baseline values of our outcome measures reflect indications for surgery. Further, the use of prospectively collected outcomes make future comparisons across clinical studies much more feasible. Moreover, patient-reported outcomes in neurosurgical research are often lacking and may provide a better understanding of the effectiveness and safety of surgical procedures [7]. The main limitation of the present study is that the loss to follow-up was relatively high. However, in a study on an equivalent patient population with 22% non-responders, no difference in outcomes between responders and non-responders was found at long-term follow-up [18].

Also, ideally we would have a control group undergoing conservative management. However, based on the symptom duration, it is unlikely that adolescents are fast-tracked to surgery, and faster recovery and surgical treatment is reserved for acute or intolerable pain, or when conservative treatment fails. Similar to SweSpine, the NORspine registry covers degenerative spine surgery as a whole and consequently no validated adolescent outcome measures were used [12]. Although we found no difference at 1 year, a longer follow-up may be warranted to investigate surgery rates for disc reherniation and detect progression of symptoms and back-pain-related disability.

## Conclusions

At 1 year, the effectiveness and safety of one level microdiscectomy are similar in adolescents and the adult population.
